# Tuberculosis Associated Chylothorax: A Case Report

**DOI:** 10.1002/rcr2.70107

**Published:** 2025-02-03

**Authors:** Niharika Malego, Ranjan Sapkota, Aakriti Sharma, Pratikshya Thapaliya

**Affiliations:** ^1^ Department of Cardio‐Thoracic and Vascular Surgery Manmohan Cardiothoracic Vascular and Transplant Center, Institute of Medicine, Tribhuvan University Kathmandu Nepal

**Keywords:** chylothorax, surgical management, thoracic duct ligation, thoracic surgery, tuberculosis

## Abstract

Chylothorax is the accumulation of chyle in the pleural cavity. Tuberculosis is one of its rarest causes. Diagnosis of chylothorax can be made by evaluation of triglycerides concentration in pleural fluid. It is primarily managed medically but surgery is indicated when the patients fail to improve after a trial of medical management. Here, we present a case of a 28‐year man who developed chylothorax while undergoing anti‐tuberculous treatment (ATT). After failing initial medical management, he underwent thoracic duct ligation with good result.

## Graphical abstract

This is a case report on a TB associated chylothorax who underwent thoracic duct ligation.

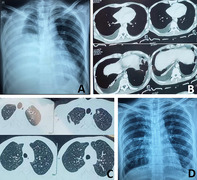

## 1 Introduction

Tuberculosis, especially tuberculous lymphadenopathy, is an extremely rare cause for chylothorax [1]. Majority of chylothorax patients respond to medical management, whereas surgery is required for those which are massive, prolonged or not responding to conservative management. Here we present a successful surgical management of a case of chylothorax associated with lymph nodal tuberculosis.

## 2 Case Report

A 28‐year‐old man, a cook for many years, presented with a history of non‐productive cough for a month. He also had a low‐grade fever and shortness of breath for 2 days, as well as a history of unintentional weight loss and loss of appetite for 2 months. Examination showed decreased breath sounds on the right side, and there were no additional sounds. He had been vaccinated against tuberculosis immediately after birth, as also evidenced by arm scar.

**FIGURE 1:**
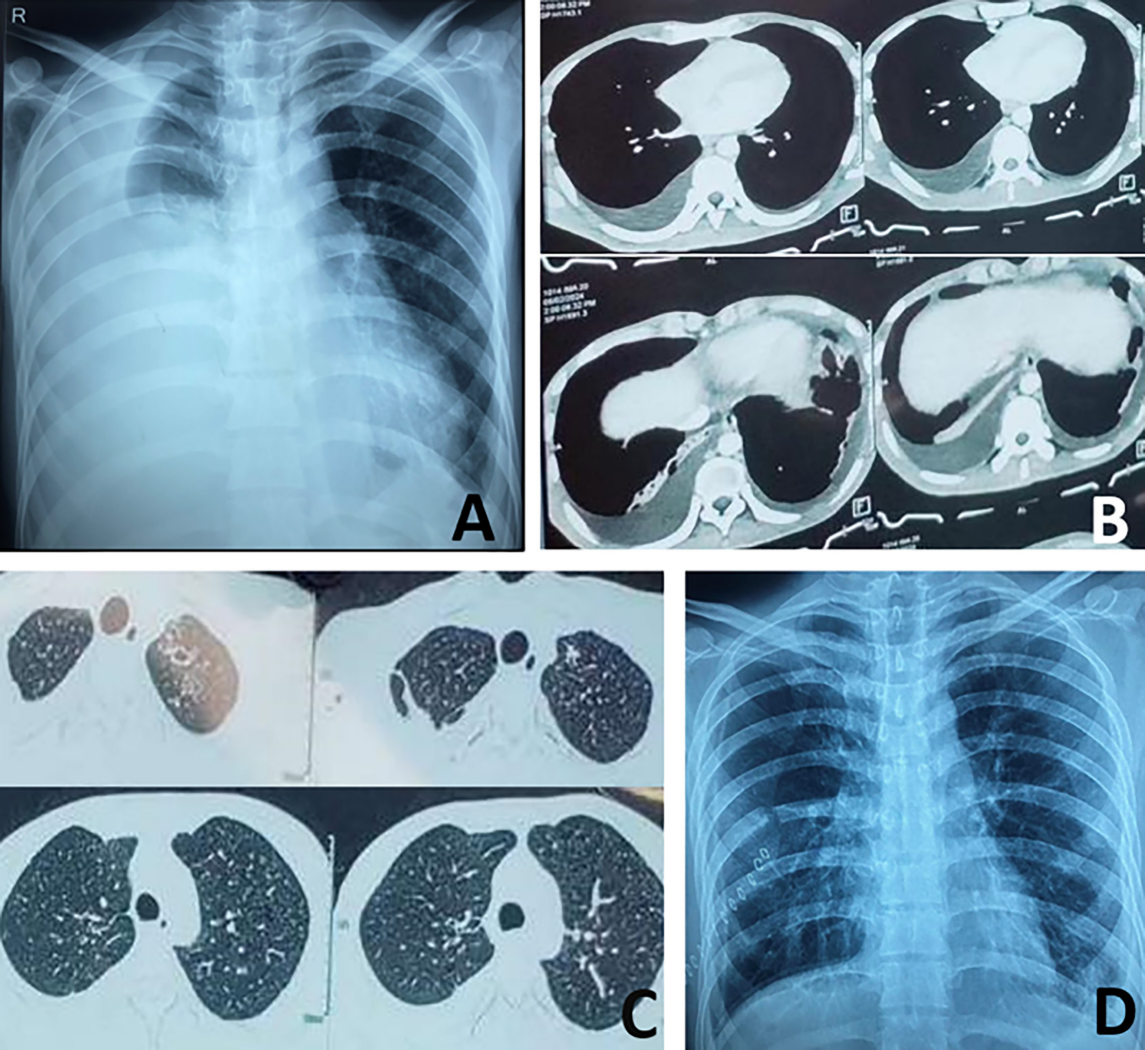
Chest x‐ray before surgery (A) and at discharge (D). Moderate pleural effusion without obvious mediastinal or pleural pathology (B); Small cavitation noted in left upper lobe and bilateral miliary nodules (C).

**FIGURE 2:**
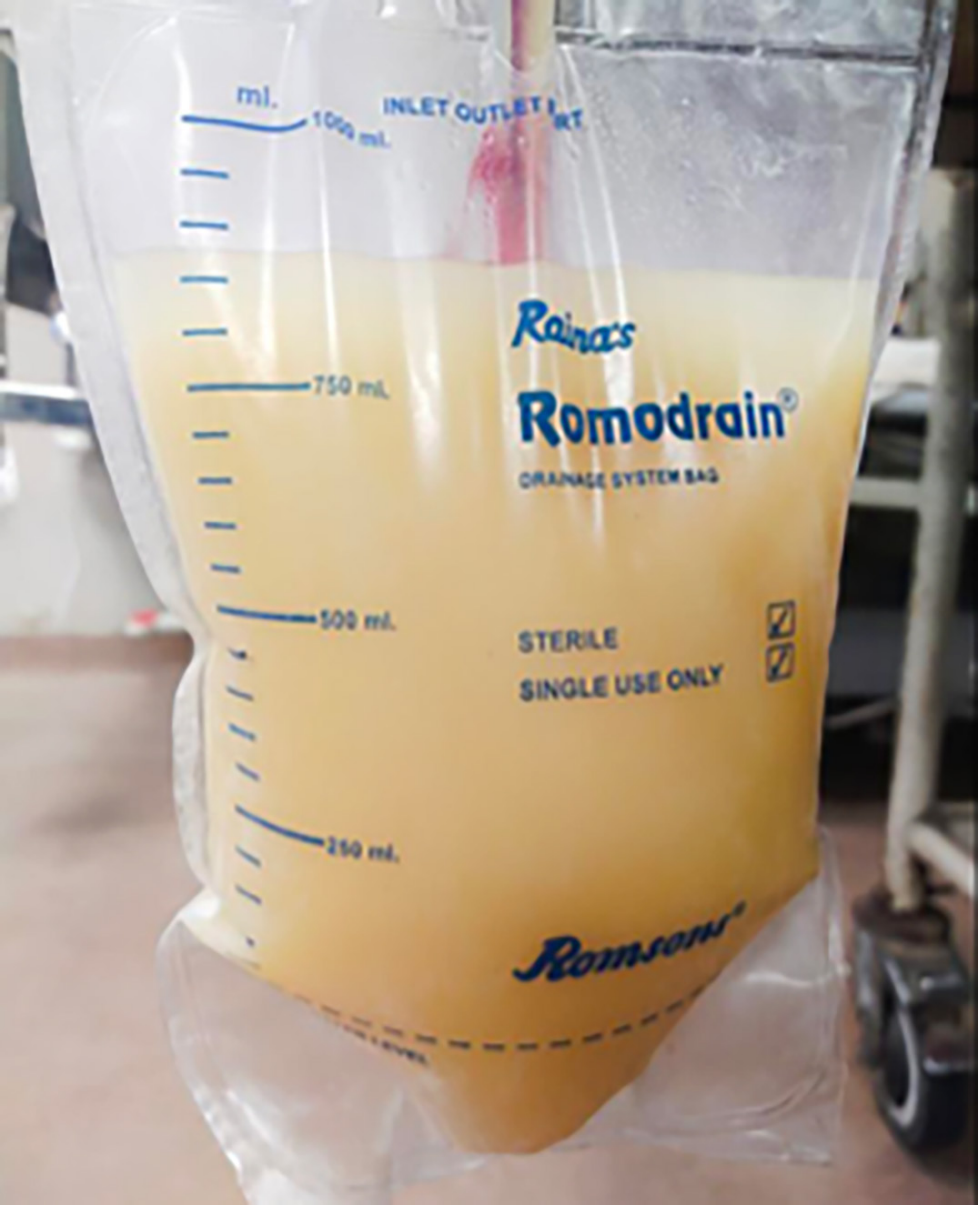
Milky white fluid in the under‐water seal bag at admission.

He had initially visited a hospital in India where he worked, and there he had been diagnosed with right‐sided pyothorax secondary to extrapulmonary tuberculosis (EPTB). At that time, sputum microscopy had not shown acid‐fast bacilli (AFB), and tuberculosis cultures were not sent for. He was managed with chest tube drainage for 3 days and empirical ATT. Initial brief clinical improvement was interrupted by shortness of breath 2 weeks later. New chest radiograph showed a recurrent pleural effusion on the same side, and a chest tube was re‐inserted (Figure 1). The fluid was milky white; and the total leukocyte count was 400/mm^3^ (20% polymorphs and 80% monomorphs); glucose concentration was 5.5 mmol/L; protein concentration was 21 g/L; and triglyceride concentration was 743 mg/dL. The diagnosis of chylothorax was thus confirmed, and octreotide infusion was started, while continuing ATT. The daily drain output was more than 2 L, and subsequently he was referred to our center (Figure 2). He was kept on total parenteral nutrition (TPN), but with no apparent improvement in drainage output. As it was already a week of high‐volume drainage, a decision was made to offer him a surgical treatment. The patient was fed 100 mL of olive oil orally 2 h prior to intubation to facilitate precise identification of lymphatic leakage.

Under general anaesthesia with double‐lumen endotracheal intubation, the right pleural cavity was explored via a right minithoracotomy at the 6th intercostal space. After the residual chyle was evacuated, large lymph nodes measuring more than 2 cm were seen in the lower posterior mediastinum, compressing the thoracic duct which was grossly dilated and pouring chyle continuously from the point of rupture a few centimetres above the hiatus. The thoracic duct was doubly ligated both proximally and distally, and a lymph node was excised for histological examination. No other leaks were identified after provocative Valsalva manoeuvres. There were no pleural deposits, and the lung was normal. Intraoperative bronchoscopy was normal.

The patient recovered well after surgery. Oral intake was started and gradually escalated to a normal diet. There was a steady reduction in the drainage output to less than 200 mL/day although the patient was on a fat‐rich diet. As the chest radiography finding was normal, the tube was removed on the fifth postoperative day. Lymph node (LN) examination showed aggregated histiocytic cells, epithelioid cells and small amount of caseous necrosis with dense infiltration by lymphocytes and plasma cells; Ziehl‐Nelsen stain showed few acid‐fast bacilli (AFB). A polymerase chain reaction (PCR) testing of the necrotic pulp of the lymph node showed “ *Mycobacterium tuberculosis* detected‐medium).” The bronchoalveolar lavage as well as the chylous fluid were negative for AFB. The chylous fluid was not sent for PCR or cultures.

The patient recently completed a 6‐month course of ATT as per Directly Observed Treatment, Short‐Course (DOTS), uneventfully. There were no pathological findings on follow‐up examination at 6 months.

## 3 Discussion

Chylothorax is the accumulation of chyle in the pleural cavity [1]. Chyle refers to the milk‐like fluid consisting of triglycerides in the form of chylomicrons, T lymphocytes, electrolytes, proteins, immunoglobulins and fat‐soluble vitamins, which is carried by the thoracic duct from the intestines and lower body to the bloodstream [2]. The exudative pleural fluid of a chylothorax will have a concentration of triglycerides over 110 mg/dL and cholesterol below 200 mg/dL, and a cholesterol: triglyceride ratio of less than one [3].

Commonest causes are trauma and malignancy, lymphoma in particular [1, 4]. Tuberculosis is an extremely rare cause of chylothorax. Mediastinal adenopathy compressing the thoracic duct, leading to its dilatation and subsequent rupture causing leakage of chyle into the pleural space, has been suggested as the pathogenesis for TB‐associated chylothorax [5], like in our case with the large lymph node. Abdominal lymphadenopathy occluding the cisterna chyli leading to the formation of lymphaticovenous anastomosis [6]; and constrictive pericarditis leading to reduced drainage [5], have also been reported. One such study reported mediastinal lymph nodes to be the cause in half of all cases, also highlighting the need for biopsy of node or pleura [7]. Chylothorax has also been reported in some cases after initiation of ATT, such as in our case wherein the patient developed chylothorax 2 weeks after initiation of ATT. The mechanism in such instances has been proposed to be an immune‐mediated thoracic duct damage [7, 8].

Treatment for tuberculous chylothorax includes ATT, a low‐fat diet, adequate drainage and Octreotide, like in our patient. Surgery is indicated when there is no improvement after 5 days of conservative management or chyle loss is > 1500 mL over 24 h [2, 7, 9]. Therefore, the patient underwent thoracic duct ligation after which there was no more chylous drainage. The patient went on to complete ATT as per the protocol, and recovered completely. In conclusion, the combination of surgical and medical management is an effective therapeutic strategy for this pathology.

## Author Contributions

**Niharika Malego and Ranjan Sapkota:** conception and design, writing, data curation and editing. **Aakriti Sharma and Pratikshya Thapaliya:** investigation, resources, and review. All authors have read and approved the final version of the manuscript.

## Ethics Statement

The authors declare that appropriate written informed consent was obtained for the publication of this manuscript and accompanying images.

## Conflicts of Interest

The authors declare no conflicts of interest.

## Data Availability

Data sharing not applicable to this article as no datasets were generated or analysed during the current study.
